# Pulmonologists' Perspectives on and Access to Palliative Care for Patients With Idiopathic Pulmonary Fibrosis in South Carolina

**DOI:** 10.1089/pmr.2023.0038

**Published:** 2023-10-30

**Authors:** Kathleen Oare Lindell, Mohan Madisetti, Tracy Fasolino, MaryChris Pittman, Patrick Coyne, Timothy P.M. Whelan, Martina Mueller, Dee W. Ford

**Affiliations:** ^1^ College of Nursing, Medical University of South Carolina, Charleston, South Carolina, USA.; ^2^ Division of Pulmonary and Critical Care Medicine, College of Medicine, Medical University of South Carolina, Charleston, South Carolina, USA.; ^3^ School of Nursing, College of Behavioral, Social, & Health Sciences, Clemson University, Clemson, South Carolina, USA.

**Keywords:** Idiopathic Pulmonary Fibrosis, palliative care, pulmonologists, serious illness

## Abstract

**Background:**

Idiopathic pulmonary fibrosis (IPF) is a serious illness with an unpredictable disease course and survival rates comparable with some cancers. Patients with IPF suffer considerable symptom burden, declining quality of life, and high health care resource utilization. Patients and caregivers report many unmet needs, including a desire for more education regarding diagnosis and assistance with navigating disease trajectory. Compelling evidence suggests that palliative care (PC) provides an extra layer of support for patients with serious illness.

**Research Question:**

The purpose of this survey was to gain perspectives regarding PC for patients with IPF by board-certified pulmonologists in South Carolina (SC).

**Study Design and Methods:**

A 24-item survey was adapted (with permission) from the Pulmonary Fibrosis Foundation PC Survey instrument. Data were analyzed and results are presented.

**Results:**

Pulmonologists (*n* = 32, 44%) completed the survey; 97% practice in urbanized settings. The majority agreed that PC and hospice do not provide the same service. There were varying views about comfort in discussing prognosis, disease trajectory, and addressing advance directives. Options for ambulatory and inpatient PC are limited and early PC referral does not occur. None reported initiating a PC referral at time of initial IPF diagnosis.

**Interpretation:**

Pulmonologists in SC who participated in this survey are aware of the principles of PC in providing comprehensive care to patients with IPF and have limited options for PC referral. PC educational materials provided early in the diagnosis can help facilitate and guide end-of-life planning and discussions. Minimal resources exist for patients in underserved communities.

## Introduction

Idiopathic pulmonary fibrosis (IPF), a progressive lung disease of aging, affects ∼250,000 people in the United States, with 50,000 new cases diagnosed each year.^1^ IPF is a serious illness with an unpredictable disease course^2^ with survival rates comparable with some aggressive cancers.^3^ Despite the advances that slow disease progression, most patients with IPF succumb to their disease or require lung transplant within five years of diagnosis.^4^

Patients newly diagnosed with IPF, and their caregivers must quickly adapt to lives dominated by a chronic progressive disease. For the patient, this is associated with declining physical function, constraints of supplemental oxygen, social isolation, impaired quality of life, shortened lifespan, and high health care resource utilization.^5^ A diagnosis of IPF is burdensome for the caregiver as well, who may feel an overwhelming sense of fear of the unknown and often, due to age, has their own comorbidities to manage.^6^ Patients and their caregivers report many unmet needs, including a desire for more education regarding their diagnosis and assistance with navigating disease trajectory.^7,8^

International and national guidelines recommend that palliative care (PC) be offered to patients who are diagnosed with serious illnesses, including IPF.^2,9–11^ Compelling evidence suggests that PC provides an extra layer of support while patients with serious illness receive nonpharmacological and pharmacological therapies.^12^ Despite an extensive body of literature that supports PC as standard of care in patients with serious conditions,^13,14^ a major gap in the literature reveals that referral to PC for patients with advanced lung disease, including IPF and lung cancer, commonly occurs late or not at all.^15–19^

**FIG. 1.**
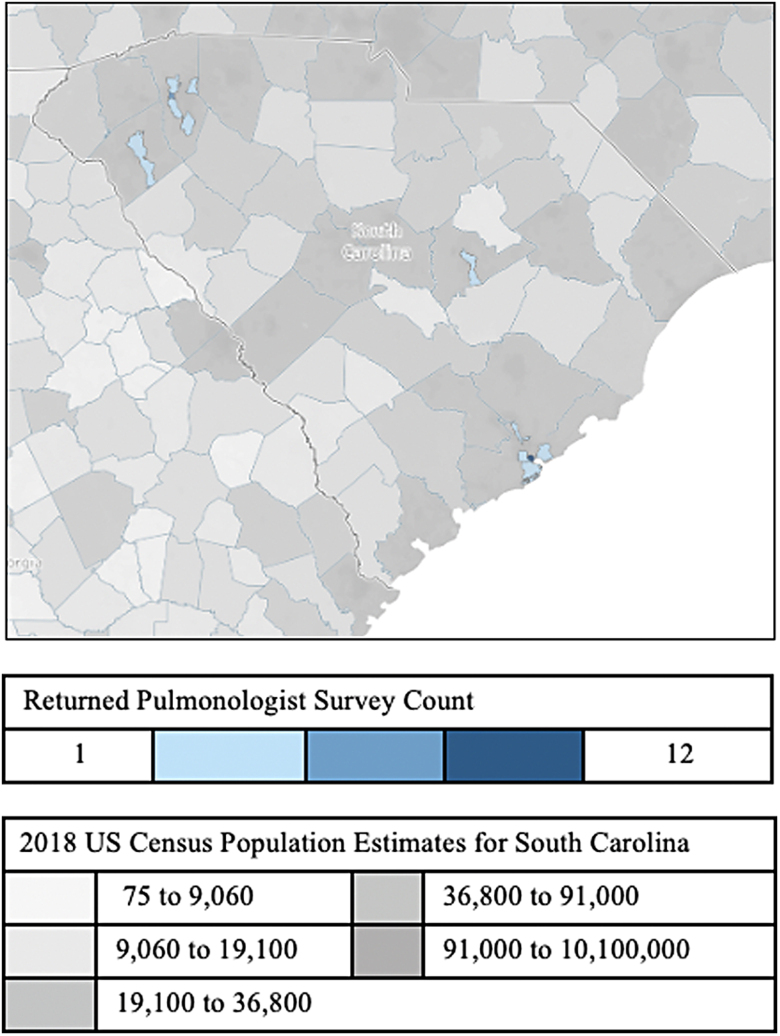
Geographic distribution of returned surveys by pulmonologists' practice zip code and state level (SC) 2018 U.S. Census population estimates. SC, South Carolina.

Social determinants of health (SDOH) further complicate access to care and neighborhood disadvantage negatively impacts mortality.^20^ Disparities related to health care access, particularly for those residing in medically underserved areas and/or rural communities, create barriers to health equity.^21^ Located on southeastern U.S. coast, South Carolina (SC) has a population of 5,282,634 residents: White 68.8%, Black 26.7%, and Hispanic 6.4%, and is predominantly rural with 40 of its 46 counties defined as rural by the U.S. Census (Fig. 1).^22^

With an estimated population of 170 residents per sq. mile, more than 95% of the population live in a Primary Care Health Professional Shortage Area and 17.6% live in poverty. It is difficult to determine the exact incidence and prevalence of IPF in SC, but chronic lower respiratory disease is the fifth leading cause of death^23^ in the state at a rate of 43.6 deaths per 100,000, ranking SC as the 11th highest state in the United States. The most recent data for the state of SC published in December 2019 suggest that, although it is difficult to quantify the ongoing burden of serious illness in the state, of the South Carolinians who died in 2018 an estimated 53.8% would have been eligible for PC but did not receive it.^24^

In 2021, the Pulmonary Fibrosis Foundation (PFF) conducted a survey^25^ among 128 health care providers across 68 PFF Care Center Network sites^26^ in the United States about current perspectives on PC for pulmonary fibrosis (PF) patients and barriers to PC for this patient population. In conclusion, they found most providers utilize PC, but there is lack of established optimal timing for PC referral.^25^ Furthermore, this study was conducted in specialty PFF Centers and thus may not represent the perspectives of community practicing pulmonary physicians.

The purpose of this survey was to gain pulmonologists' perspectives on and access to PC for patients with IPF and their caregivers in SC, a rural state with significant health care disparities.^27^

## Study Design and Methods

### Setting and participants

This study was conducted among board-certified pulmonologists who practice in SC and care for patients with IPF. The current workforce of pulmonologists in SC lists 72 pulmonologists with 71,510 people per physician, ranking 36th in the United States for active pulmonologists per 100,000 population.^28^

### Survey instrument

The 24-item survey was adapted (with permission) from the PFF PC Survey^25^ instrument and converted by the researchers into a Research Electronic Data Capture (REDCap) survey for online dissemination.

### Data collection and management

This study used REDCap hosted on secure institutional servers to disseminate the survey, collect, and manage data. All pulmonologists in SC^28^ were sent a REDCAP survey invitation link through e-mail using features that prevented linking pulmonologists to their responses. Pulmonologists received four-monthly automated system reminders to complete the survey. Data were collected from June to September 2022.

### Statistical analysis

Data were analyzed using Microsoft Excel 16.6. Results are presented using basic descriptives (frequencies and percentages) to summarize findings. The frequencies of missing returned data are presented in the data tables; however, they are excluded from the final analysis.

### Ethical considerations

Per Medical University of South Carolina (MUSC) Institutional Review Board (IRB), this project was not subject to IRB review or approval. All participants were informed of the voluntary nature of survey completion and could refuse to respond to any question or stop participation at any time. Given the same sample size of registered pulmonologists in SC and the potential for the identification of individuals and/or respective employers based on responses, participant's race and ethnicity data are not presented, and practice zip code data has been aggregated in reporting to protect their privacy and confidentiality. Study compensation was not provided to participants for survey completion.

## Results

Seventy-two board-certified pulmonologists practice in SC; 44% (32/72) completed the survey. The majority (75%) of these respondents reported more than five years of experience in providing care to IPF patients (Table 1) and ∼97% practice in cities (>2500 people) and 3% in rural areas (<2500 people) of SC, respectively (Fig. 1). Most could differentiate PC and hospice. Just more than one third of respondents were very comfortable discussing prognosis, disease trajectory, and readiness for PC.

**Table 1.** Pulmonologist Eligibility Screen

Respondent characteristics (***N*** = 32)	%	*n/N*
Years of experience in providing IPF care		
0–5 years	25.0	8/32
6–10 years	28.1	9/32
11–15 years	18.8	6/32
>15 years	28.1	9/32

Practice setting		
Metropolitan area (100,000 + people)	56.3	18/32
Urbanized area (50,000–99,999 people)	31.3	10/32
Urbanized cluster area (2500–49,999 people)	9.4	3/32
Rural area (<2500 people)	3.1	1/32

IPF, idiopathic pulmonary fibrosis.

Less than one third of the respondents strongly agreed that they addressed advance directives with patients. The majority evaluated symptoms quarterly, however, did not report use of a standardized instrument to assess symptom burden. Less than one quarter of respondents strongly agreed that they used PC with varied results on availability of PC resources and timing of referral. Lastly, the majority reported that handouts describing the disease and PC options for IPF patients and caregivers would be helpful (Table 2).

**Table 2.** South Carolina Pulmonologist's Survey Results

Survey items	Total respondents ***N*** = 32
Pulmonologist baseline knowledge of PC and hospice services	
PC and hospice offer the same service(s)	
Strongly disagree	9 (28.1)
Disagree	21 (65.6)
Neutral	0 (0)
Agree	2 (6.3)
Strongly agree	0 (0)
PC services include: (check all that apply)	
Symptom management	32 (100.0)
Advance directives	31 (96.9)
Spiritual care	31 (96.9)
Psychological care	30 (93.8)
Pulmonologist comfort in communication discussing prognosis and disease trajectory, patient's readiness for and acceptance of PC and important of advance directives	
I am comfortable discussing prognosis and disease trajectory with my IPF patients.	
Strongly disagree	0 (0)
Disagree	1 (3.1)
Neutral	1 (3.1)
Agree	18 (56.3)
Strongly agree	12 (37.5)
I feel comfortable assessing a patient's readiness for and acceptance of PC.	
Strongly disagree	0 (0)
Disagree	0 (0)
Neutral	6 (18.8)
Agree	16 (50.0)
Strongly agree	10 (31.3)
It is important to address advance directives with my IPF patients.	
Strongly disagree	0 (0)
Disagree	0 (0)
Neutral	2 (6.3)
Agree	11 (34.4)
Strongly agree	19 (59.4)
I address advance directives with my IPF patients.	
Strongly disagree	0 (0)
Disagree	1 (3.1)
Neutral	5 (15.6)
Agree	17 (53.1)
Strongly agree	9 (28.1)
Pulmonologist practices regarding symptom burden assessment, use of symptom assessment questionnaires, and frequency of performed symptom monitoring	
What symptoms do you routinely monitor your IPF patients for? (Check all that apply)	
Dyspnea	27 (90.0)
Cough	26 (86.7)
Depression	16 (53.3)
Anxiety	14 (46.7)
Fatigue	20 (66.7)
Exercise tolerance	26 (86.7)
General well-being	21 (70.0)
Not performed	1 (3.3)
Do you use specific tools, questionnaires, or procedures to monitor any of these symptoms?	
No	23 (76.7)
Yes	7 (23.3)
Missing^a^	2
Who is responsible for symptom management where you practice?	
Nurse	1 (3.3)
APP	1 (3.3)
Physician	23 (76.7)
Other	5 (16.7)
Missing^a^	2
How frequently are symptoms monitored?	
Not performed	3 (10)
Prefer not to say	1 (3.3)
Quarterly	23 (76.7)
Yearly	3 (10)
Pulmonologist practices related to PC referrals	
I utilize PC services for my patients with IPF.	
Strongly disagree	0 (0)
Disagree	4 (13.3)
Neutral	4 (13.3)
Agree	15 (50.0)
Strongly agree	7 (23.3)
Missing^a^	2
I initiate a PC referral for my patients with IPF. (Check all that apply)	
At initial ILD/IPF diagnosis	0 (0.0)
Objective disease progression	11 (36.7)
Symptomatic progression	20 (66.7)
Hospitalization	14 (46.7)
I rarely refer patients to PC	3 (10)
Missing^a^	2
If you responded “I rarely refer patients to Palliative Care”… is this due to…	
Lack of PC services at your institution	1 (33.3)
Insufficient time in clinic to discuss PC	1 (33.3)
I am comfortable addressing PC needs in my practice	1 (33.3)
PC services are available at my institution: (check all that apply)	
Within my practice	5 (16.7)
Subspecialty outpatient clinic	5 (16.7)
For Inpatients only	10 (33.3)
For inpatients and outpatients	17 (56.7)
Not available	0 (0.0)
Prefer not to say	1 (3.3)
Missing^a^	2
Pulmonologist practices regarding the use of PC assessment tools and need for educational materials for patients and caregivers	
We routinely assess health related quality of life with a standardized questionnaire in our practice.	
Strongly disagree	0 (0)
Disagree	26 (86.7)
Neutral	1 (3.3)
Agree	0 (0)
Strongly agree	0 (0)
Prefer not to say	3 (10.0)
Missing^a^	2
We routinely assess symptom burden with a standardized questionnaire in our practice.	
Strongly disagree	0 (0)
Disagree	26 (86.7)
Neutral	0 (0)
Agree	1 (3.3)
Strongly agree	0 (0)
Prefer not to say	3 (10.0)
Missing^a^	2
A handout describing PC options for IPF patients and caregivers would be helpful in our practice.	
Strongly disagree	0 (0)
Disagree	1 (3.3)
Neutral	3 (10.0)
Agree	14 (46.7)
Strongly agree	12 (40.0)
Missing^a^	2
A handout describing long term prognosis and trajectory in IPF would be helpful in my practice.	
Strongly disagree	0 (0)
Disagree	2 (6.7)
Neutral	4 (13.3)
Agree	10 (33.3)
Strongly agree	14 (46.7)
Missing^a^	2

^a^ Missing data not included in analysis.

APP, advanced practice provider; PC, palliative care.

## Discussion

The purpose of this survey was to gain pulmonologists' perspectives on and access to PC for patients with IPF and caregivers in SC, a rural state with significant health care disparities.^27^ Survey findings reveal that these pulmonologists had knowledge that PC and hospice offered different services. Comfort in communication practices differed for these respondents. Particularly concerning in the results are the relatively small proportions (∼30%) of practicing pulmonologists that reported strongly agreeing with their comfort level discussing prognosis and referral to PC with their IPF patients.

If a patient's pulmonologist does not feel proficient in having these difficult conversations, the question begs will this conversation occur and who will have it. While pulmonologists endorsed querying patient's symptom burden and quality of life, very few used structured tools to accomplish this goal. This is concerning that respondents may not be systematically evaluating patient symptom burden, including anxiety and depression, and related treatment options. The majority had access to some level of PC, but referral to PC was reported not to occur early in the disease course for patients with IPF. Approximately one-half of pulmonologists (47%) refer to PC when the patient is hospitalized.

These findings support past work about unmet patient needs^7,8^ and delayed referral for PC.^19,29,30^ There are several reasons why patients with IPF are not referred to PC or referred late in the disease course. These include limited time during the office visit to have this serious illness discussion, many providers are uncomfortable with PC and fear that such discussions will diminish hope, and limited PC resources.^31^

The serious nature of IPF, compounded by the unpredictable disease course, mandates that serious illness communication should occur early after diagnosis to promote symptom management and identify patient's wishes as they address end of life (EOL).^32^ Patients whose prioritized health care goals are understood by their clinicians are likely to be better positioned to receive goal-concordant care, defined as “medical care that aligns with the patients' values, goals, and life priorities.^33,34^ PC provides this extra layer of support while patients with serious illness receive nonpharmacological and pharmacological therapies throughout their disease course.^12^

Sullivan et al. propose a model for collaborative PC in serious respiratory illness with a joint collaboration between pulmonologists and primary and specialty PC providers early in the disease.^35^ Although our study did not query pulmonologists' perception of telehealth as a tool to increase access to PC, this has been shown to be a feasible and acceptable approach and can be facilitated through telehealth delivery to patients and caregivers earlier in the disease course, including those in underserved and/or rural communities.^36^ Remote monitoring of patients enrolled in PC through telehealth has been shown to be acceptable and feasible.^37^

Addressing SDOH that compound barriers in access to PC for patients with IPF and caregivers is of high importance. Similar to many states, SC is largely rural, where many patients travel a significant distance to receive specialty care to confirm diagnosis and treatment plan. In addition, many of these patients lack reliable transportation compounding SDOH. These patients then frequently return to remote areas that lack resources, including partners in care, thus leading to health care deprivation and social isolation. The most deprived counties cluster in rural parts of SC, whereas the more affluent counties cluster in the more urban parts of the state. It is important to note that there are urban areas in SC that are underserved.^22^ In previous study, we found that patients with IPF living in neighborhoods with greater disadvantage experienced higher mortality.^20^

### Limitations

This survey was conducted among board-certified pulmonologists in SC and may not be reflective of the care patients with IPF receive in PFF Centers. Although we adapted and disseminated a survey instrument that had previously been devised by an expert panel and used to explore IPF provider's perceptions on a national level, the instrument has not yet been validated. Owing to the small sample size, the observed 44% (32/72) response rate may limit the generalizability of our findings.

This survey would benefit from replication in other states with similar rural geography to look for similarities or differences in practice. In addition, although this survey assessed pulmonologist's perceptions of and access regarding PC in our state, there is no patient-level data to determine what happens from the patient/caregiver voice. As mentioned earlier, of the South Carolinians who died in 2018 an estimated 53.8% would have been eligible for PC but did not receive it. This report does not break down by disease category or practitioner type.^24^

### Interpretation

Pulmonologists in SC who participated in this survey are aware of the principles of PC in providing comprehensive care to patients with IPF. Symptom management was viewed to be important but widespread use of standardized tools to measure symptom burden is uncommon. This is understandable in a busy clinical practice where the focus is on the physician/patient interaction but raises concerns about unmet symptom assessment and management needs. Educational resources are needed to increase the pulmonologists' comfort in facilitating conversations around PC as well as help explain the disease trajectory early in diagnosis, and to facilitate EOL care planning (e.g., advance directives).

With limited options for PC referrals, we endorse use of other avenues for early delivery of PC including national PC resources where providers and patients can access information about PC, for example, Get.PC.com: https://getpalliativecare.org/^38^ and PFF PC Position Statements for Providers and Patients.^39,40^

## Take-Home Points

Communication of serious illness is critical for patients diagnosed with IPF and caregiver(s).

Practicing pulmonologists should seek proficiency in having these serious illness discussions regarding prognosis and referral to PC for IPF patients.

SC workforce of pulmonologists ranks 36th in the nation; this workforce shortage may contribute to delay in PC discussion.

PC resources are available to guide patients and caregivers to independently seek out PC.

Telehealth delivery of PC and nurse-led programs can address the SDOH and access to PC.

## Abbreviations Used

APP: advanced practice provider
EOL: end of life
ICLAF: International Colloquium of Lung and Airway Fibrosis
ICMJE: International Committee of Medical Journal Editors
IPF: idiopathic pulmonary fibrosis
IRB: Institutional Review Board
MUSC: Medical University of South Carolina
PC: palliative care
PF: pulmonary fibrosis
PFF: Pulmonary Fibrosis Foundation
REDCap: Research Electronic Data Capture
SC: South Carolina
SCTA: South Carolina Telehealth Alliance
SCTR: South Carolina Clinical & Translational Research
SDOH: social determinants of health
